# Postoperative Chemoradiotherapy With Capecitabine and Oxaliplatin vs Capecitabine for Stage II to III Rectal Cancer: A Randomized Clinical Trial

**DOI:** 10.1001/jamanetworkopen.2021.36116

**Published:** 2021-11-30

**Authors:** Ning Li, Yuan Zhu, Lu-Ying Liu, Yan-Ru Feng, Wen-Ling Wang, Jun Wang, Hao Wang, Gao-Feng Li, Yuan Tang, Chen Hu, Wen-Yang Liu, Hua Ren, Shu-Lian Wang, Wei-Hu Wang, Yong-Wen Song, Yue-Ping Liu, Hui Fang, Yu Tang, Ning-Ning Lu, Bo Chen, Shu-Nan Qi, Xin-Fan Liu, Ye-Xiong Li, Jing Jin

**Affiliations:** 1 State Key Laboratory of Molecular Oncology, Department of Radiation Oncology, National Cancer Center/National Clinical Research Center for Cancer/Cancer Hospital, Chinese Academy of Medical Sciences, Peking Union Medical College, Beijing, China; 2 Department of Radiation Oncology, Cancer Hospital of the University of Chinese Academy of Sciences, Zhejiang Cancer Hospital, Institute of Cancer and Basic Medicine, Chinese Academy of Sciences, Hangzhou, China; 3 Department of Radiation Oncology, Guizhou Cancer Hospital, Guiyang, China; 4 Department of Radiation Oncology, Tumor Hospital of Hebei Province, Shijiazhuang, China; 5 Department of Radiation Oncology, Peking University Third Hospital, Beijing, China; 6 Department of Radiation Oncology, Beijing Hospital, Beijing, China; 7 Division of Biostatistics and Bioinformatics, Sidney Kimmel Comprehensive Cancer Center, Johns Hopkins University School of Medicine, Baltimore, Maryland; 8 Department of Radiation Oncology, Beijing Cancer Hospital, Beijing, China

## Abstract

**Importance:**

Several studies have explored the efficacy and toxic effects of concurrent 5-fluorouracil (5-FU)– or capecitabine-based chemoradiotherapy (CRT) with or without oxaliplatin in the neoadjuvant setting. Addition of oxaliplatin to 5-FU or capecitabine elicited similar outcomes but with significantly increased toxic effects; however, there is a need for randomized clinical trials comparing 2 CRT regimens for patients receiving CRT in the adjuvant setting.

**Objective:**

To explore the efficacy and toxic effects of oxaliplatin combined with postoperative concurrent capecitabine and radiotherapy (RT) for pathological stage II and III rectal cancer.

**Design, Setting, and Participants:**

This multicenter randomized clinical trial enrolled patients from 7 centers in China between April 1, 2008, and December 30, 2015. Patients with pathologically confirmed stage II and III rectal cancer were randomized (1:1) to receive concurrent CRT with capecitabine or capecitabine plus oxaliplatin. Analysis was conducted from December 31, 2019, to March 15, 2020.

**Interventions:**

RT comprised 45 to 50 Gy in 25 fractions of 1.8 to 2.0 Gy over 5 weeks. In the capecitabine with RT group, concurrent chemotherapy included 2 cycles of capecitabine (1600 mg/m^2^) on days 1 to 14 and 22 to 35. The capecitabine and oxaliplatin with RT group received identical postoperative RT to that in the capecitabine with RT group combined with capecitabine (1300 mg/m^2^) on days 1 to 14 and 22 to 35 and a 2-hour infusion of oxaliplatin (60 mg/m^2^) on weeks 1, 2, 4, and 5. Patients in both groups received adjuvant chemotherapy (capecitabine or fluorouracil and oxaliplatin) after CRT.

**Main Outcomes and Measures:**

The primary end point was 3-year disease-free survival (DFS).

**Results:**

A total of 589 patients (median [IQR] age, 55 [47-52] years; 375 [63.7%] men and 214 [36.3%] women) were enrolled, including 294 patients randomized to the capecitabine with RT group and 295 patients randomized to the capecitabine and oxaliplatin with RT group. Median (IQR) follow-up was 68 (45-96) months. Most patients had stage III disease (574 patients [75.9%]). Three-year DFS was 76.3% for the capecitabine with RT group and 74.1% for the capecitabine and oxaliplatin with RT group, and 5-year DFS was 72.0% for the capecitabine with RT group and 71.1% for the capecitabine and oxaliplatin with RT group (hazard ratio [HR], 1.07; 95% CI, 0.79-1.44; *P* = .68). There was no significant difference between groups in overall survival (HR, 0.93; 95% CI, 0.64-1.34; *P* = .70) or local recurrence (HR, 0.61; 95% CI, 0.31-1.22; *P* = .16). More grade 3 and 4 acute toxic effects were observed in the capecitabine and oxaliplatin with RT group than in the capecitabine with RT group (114 patients [38.6%] vs 84 patients [28.6%]; *P* = .01).

**Conclusions and Relevance:**

This randomized clinical trial found that addition of oxaliplatin to capecitabine-based postoperative CRT did not improve the efficacy of treatment but increased the risk of severe acute toxic effects. This finding highlights the basic role of postoperative capecitabine with RT for patients with locally advanced rectal cancer.

**Trial Registration:**

ClinicalTrials.gov Identifier: NCT00714077

## Key Points

**Question:**

Can adding oxaliplatin to postoperative capecitabine-based chemoradiotherapy (CRT) and contemporary adjuvant chemotherapy regimens in locally advanced rectal cancer improve the efficacy of treatment?

**Findings:**

In this randomized clinical trial of 602 adults, the addition of oxaliplatin to capecitabine-based postoperative CRT did not significantly improve disease-free survival.

**Meaning:**

These findings suggest that capecitabine-based postoperative CRT could be considered to be an alternative type of multidisciplinary management of locally advanced rectal cancer for patients who did not receive neoadjuvant CRT.

## Teaser

This randomized clinical trial examines the efficacy and toxic effects of postoperative capecitabine vs oxaliplatin plus capecitabine with radiotherapy for stage II and III rectal cancer.

## Introduction

Colorectal cancer is the third most common cancer and the third leading cause of cancer-related death in the US.^1^ In China, the estimated number new cases of rectal cancer diagnosed each year is 376 300.^2^ Rectal cancer accounts for nearly one-third of colorectal cancer cases.

Historically, a combination of postoperative fluorouracil-based chemoradiotherapy (CRT) and systemic therapy has reduced the recurrence risk and improved survival for patients with locally advanced rectal cancer compared with postoperative radiotherapy (RT) alone or surgical treatment alone.^3,4^ In the last 2 decades, various preoperative RT or concurrent CRT regimens have been developed to optimize the sequence of multidisciplinary treatment and determine the most appropriate scheduling of RT and CRT.^5,6,7,8,9^

In 3 randomized clinical trials (RCTs) comparing the efficacy of preoperative with postoperative 5-fluorouracil (5-FU) or capecitabine-based CRT,^10,11,12,13^ preoperative CRT significantly improved the prevalence of locoregional recurrence free survival^10,11^ or disease-free survival (DFS)^12^ with lower treatment-related toxic effects for patients with locally advanced rectal cancer.^11,12^ However, there was no significant improvement in overall survival (OS) between patients who received preoperative CRT and those who underwent postoperative CRT.^10,11,12,13^ Although preoperative CRT is recommended as standard treatment for patients with locally advanced rectal cancer, postoperative CRT is an option for those who do not receive preoperative CRT.^14,15^ According to Surveillance, Epidemiology and End Results data from 2000 to 2016 among patients with rectal cancer who received perioperative RT, 36.3% of patients received postoperative concurrent CRT^16^; whereas in China between 2014 and 2016, most patients (>70%) received postoperative concurrent CRT.^17^

In recent years, 5-FU or capecitabine combined with other cytotoxic drugs have become the first-line or adjuvant standard chemotherapy regimen for patients with colorectal adenocarcinoma.^18,19,20^ For patients with locally advanced rectal cancer, 5-FU–based or capecitabine-based CRT achieves similar outcomes in neoadjuvant or adjuvant settings.^21^ Furthermore, several studies have explored the efficacy and toxic effects of concurrent 5-FU– or capecitabine-based CRT with or without oxaliplatin in the neoadjuvant setting.^22,23,24,25,26^ Addition of oxaliplatin to 5-FU or capecitabine elicited similar outcomes but with significantly increased toxic effects.^22,23,24,25,26^ However, to our knowledge, no RCT has compared 2 CRT regimens for patients with rectal cancer in the adjuvant setting.

To address these concerns, we initiated a phase 3 RCT in 2008 to evaluate the efficacy and toxic effects of adding oxaliplatin to capecitabine-based adjuvant CRT followed by standard chemotherapy regimens for patients with stage II or III rectal cancer. In our interim analyses,^27^ concurrent RT with capecitabine or capecitabine plus oxaliplatin resulted in similar treatment outcomes. Here, we present the mature results of this multicenter phase 3 RCT.

## Methods

### Study Design

This multicenter, randomized clinical trial was conducted in 7 institutions in China. Participants were enrolled between April 1, 2008, and December 30, 2015; follow-up ended December 31, 2019. All participants provided written informed consent before enrollment. The study protocol was approved by local ethics committee of each institution. This study followed the Consolidated Standards of Reporting Trials (CONSORT) reporting guideline.

### Study Population

As described previously in detail,^27^ patients eligible for this RCT had stage II to III (M0) rectal cancer according to the staging system set by the American Joint Committee on Cancer in 2002. Patients had undergone curative surgical procedures (total mesorectal excision) with negative resection margins (R0 resection).

### Randomization

Patients were assigned randomly to receive RT concurrently with capecitabine or capecitabine and oxaliplatin at a ratio of 1:1. Randomization was undertaken centrally at the administration office of our study center according to computer-generated randomization codes with stratification of pathological stage (II vs III). Treatment groups were not masked throughout the RCT because of different administration of treatments and schedules. The trial protocol and statistical analysis plan are presented in Supplement 1.

### Interventions

In the capecitabine with RT group, patients received postoperative concurrent CRT with capecitabine (1600 mg/m^2^) on days 1 to 14 and 22 to 35. In the experimental group, patients received postoperative concurrent CRT with capecitabine (1300 mg/m^2^) on days 1 to 14 and 22 to 35, and a 2-hour infusion of oxaliplatin (60 mg/m^2^) on weeks 1, 2, 4, and 5 (capecitabine and oxaliplatin with RT group). RT consisted of 45 to 50 Gy in 25 fractions of 1.8 to 2.0 Gy (photons at 6 MV), 5 times per week, over 5 weeks, depending on the volume of small bowel in the pelvis. The clinical target volume included the whole pelvis.^28,29^ Adjuvant chemotherapy with 4 to 6 cycles of capecitabine and oxaliplatin or 8 to 12 cycles of fluorouracil, leucovorin, and oxaliplatin was delivered in both arms 4 weeks after CRT.

### Outcomes

After completion of treatment, patients were followed up every 3 months for 2 years, then every 6 months up to 5 years, and yearly thereafter. The primary end point was DFS, which was defined as the time from randomization to the first occurrence of locoregional recurrence, distant metastasis, or death from any cause. The secondary end points were OS, local recurrence, treatment adherence, and safety. Acute toxic effects was assessed and graded according to the Common Terminology Criteria for Adverse Events version 3.0.

### Statistical Analysis

Assuming a 10% dropout rate, 570 patients (285 per group) would provide 80% power to detect 3-year DFS of 65% for the capecitabine with RT group and 75% for the capecitabine and oxaliplatin with RT group, with a 2-sided α of .05. Safety and adherence were evaluated in the per-protocol analysis after exclusion of ineligible patients. Comparison of DFS and OS between groups according to the modified intent-to-treat principle was undertaken using a 2-sided log-rank test.

Statistical analyses were carried out using SPSS statistical software version 22.0 (IBM). Hazard ratios (HRs) with 95% CIs were calculated using the Cox proportional hazards model. Survival curves were presented according to the Kaplan-Meier method. χ^2^ and Mann-Whitney *U* tests were used to compare the differences in categorical variables and continuous variables between groups. Statistical significance was set at *P* < .05. Data were analyzed from December 31, 2019, to [placeholder].

## Results

### Study Participants

**Figure 1.**
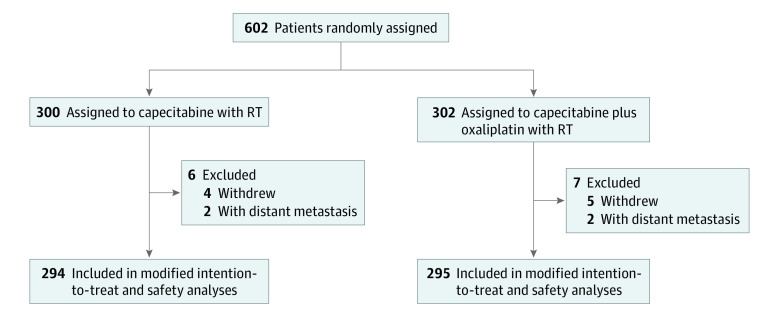
Patient Selection Flowchart RT indicates radiotherapy.

Between April 1, 2008, and December 30, 2015, 602 patients from 7 centers in China were entered into our study. Nine patients withdrew from the study, and 4 patients who had distant metastasis were excluded before adjuvant treatment. A total of 589 patients (median [IQR] age, 55 [47-62] years; 375 [63.7%] men and 214 [36.3%] women) were included in analysis, with 294 patients assigned to the capecitabine with RT group and 295 patients assigned to the capecitabine and oxaliplatin with RT group (Figure 1). Most patients had stage III disease (574 patients [75.9%]). The characteristics of patients and tumors were balanced across treatment groups (Table 1). All but 6 patients received intensity-modulated RT (IMRT; 470 patients [79.8%]) or 3-dimensional conformal RT (3D-CRT; 107 patients [18.2%]).

**Table 1.** Characteristics of Patients at Baseline

Characteristics	No. (%)	
	Capecitabine with RT (n = 294)	Capecitabine and oxaliplatin with RT (n = 295)
Sex		
Men	190 (64.6)	185 (62.7)
Women	104 (35.4)	110 (37.3)
Age, median (IQR), y	55 (47-62)	55 (47-62)
ECOG status		
0-1	288 (98.0)	284 (96.3)
≥2	6 (2.0)	11 (3.7)
Distance from anal verge, cm		
≤5	132 (45.2)	110 (37.3)
5-10	135 (45.9)	168 (56.9)
>10	27 (9.2)	17 (5.8)
pT stage		
T1-2	31 (10.5)	25 (8.5)
T3	240 (81.6)	234 (79.3)
T4	22 (7.5)	36 (12.2)
pN stage		
N0	75 (25.5)	70 (23.7)
N1	135 (45.9)	131 (44.4)
N2	84 (28.6)	94 (31.9)
pTNM stage		
II	75 (25.5)	70 (23.7)
III	219 (74.8)	225 (76.3)
Nodes retrieved, median (range), No.	17 (2-72)	17 (2-67)
Positive nodes, median (range), No.	2 (0-24)	2 (0-29)
Intravenous tumor embolus		
No	238 (81.0)	230 (78.0)
Yes	56 (19.0)	65 (22.0)
Mucinous component		
No	262 (89.1)	252 (85.4)
Yes	32 (10.9)	43 (14.6)
Surgical procedure		
Anterior resection	216 (73.4)	224 (75.9)
Abdominoperineal resection	78 (26.5)	71 (24.1)
RT method		
IMRT	230 (78.3)	240 (81.4)
3D-CRT	58 (19.7)	49 (16.6)
Conventional RT	6 (2.0)	6 (2.0)

Abbreviations: 3D-CRT, 3-dimensional conformal radiotherapy; CRT, chemoradiotherapy; ECOG, Eastern Cooperative Oncology Group; IMRT, intensity modulated radiation therapy; RT, radiotherapy.

A total of 218 patients (74.1%) in the capecitabine with RT group and 195 patients (66.1%) in the capecitabine and oxaliplatin with RT group completed the treatment protocol without dose reduction. A total of 218 patients (74.1%) in the capecitabine with RT group and 221 patients (74.9%) in the capecitabine and oxaliplatin with RT group received adjuvant chemotherapy (*P* = .17) (eTable 1 in Supplement 2). Of 150 patients who did not undergo adjuvant chemotherapy, 45 declined to take part, 10 developed disease progression before adjuvant chemotherapy, 6 had severe hematological toxic effects, and the reason was not known for 89 patients.

### Efficacy Outcomes

**Figure 2.**
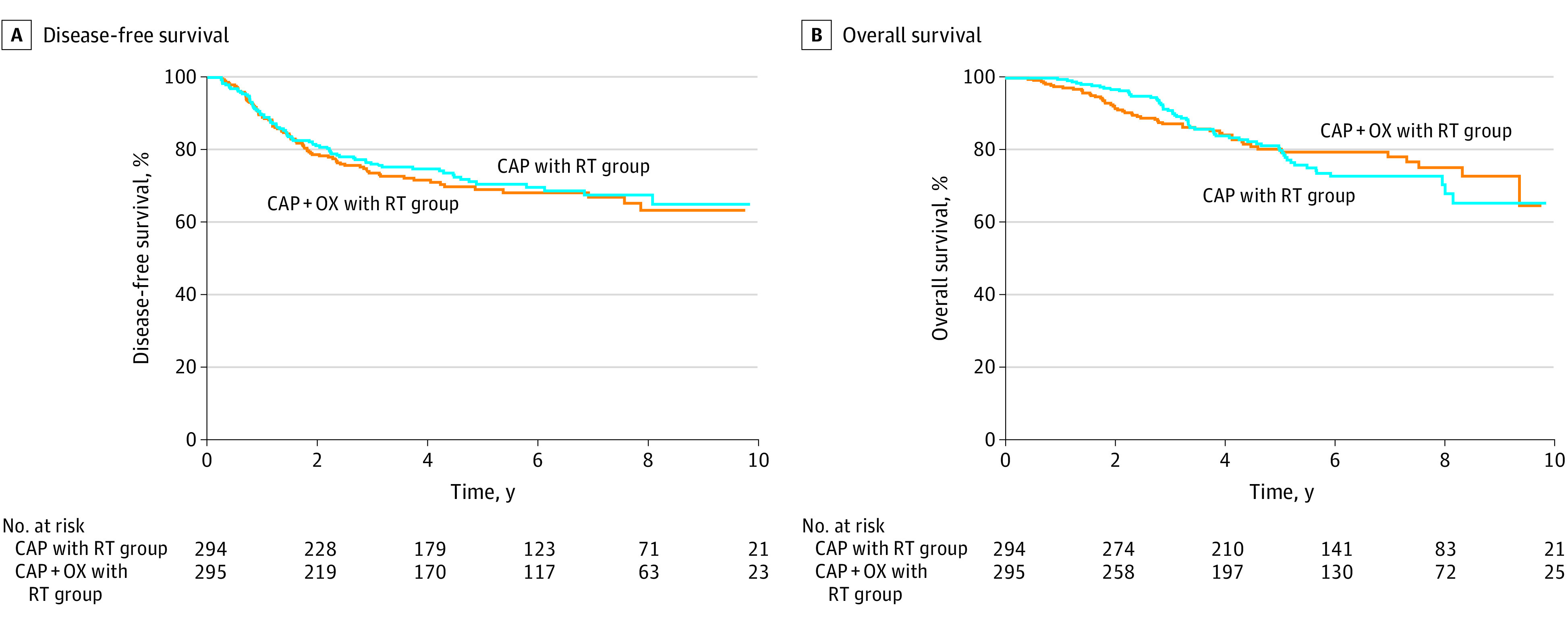
Cumulative Disease-Free Survival and Overall Survival CAP indicates capecitabine; OX, oxaliplatin; and RT, radiotherapy.

The median (IQR) duration of follow-up was 68 (45-96) months. In the capecitabine with RT group, 82 deaths or recurrence of cancer events (27.9%) occurred, and in the capecitabine and oxaliplatin with RT group, 86 deaths or recurrence events (29.1%) occurred. There was no significant difference in DFS between groups. Three-year DFS was 76.3% in the capecitabine with RT group and 72.0% in the capecitabine and oxaliplatin with RT group, and 5-year DFS was 74.1% in the capecitabine with RT group and 71.1% in the capecitabine and oxaliplatin with RT group (HR, 1.07; 95%CI, 0.79-1.44; *P* = .68) (Figure 2A).

**Figure 3.**
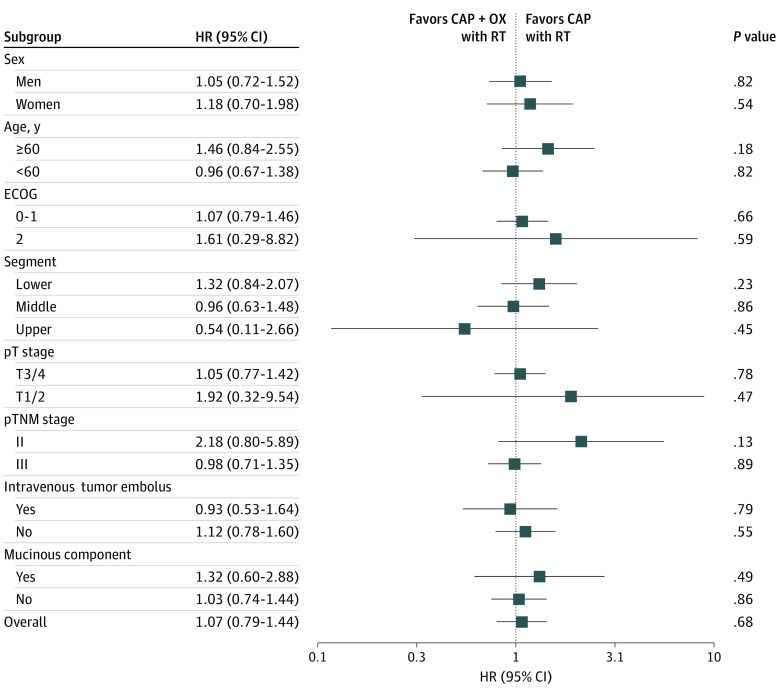
Forest Plot of Subgroup Analyses for Disease-Free Survival According to Major Prognostic Factors at Baseline Hazard ratios (HRs) for disease-free survival in subgroups of patients with rectal cancer who received postoperative capecitabine (CAP) and oxaliplatin (OX) with radiotherapy (RT) or CAP with RT. ECOG indicates Eastern Cooperative Oncology Group.

Sixty patients (20.4%) died in the capecitabine with RT group, and 53 patients (18.0%) died in the capecitabine and oxaliplatin with RT group. There was no significant difference in OS between treatment groups. Five-year OS was 82.9% for the capecitabine with RT group and 82.4% for the capecitabine and oxaliplatin with RT group (HR, 0.93; 95% CI, 0.64-1.34; *P* = .70) (Figure 2B). Five-year local recurrence-free survival was 92.9% for the capecitabine with RT group vs 95.3% for the capecitabine and oxaliplatin with RT group (HR, 0.61; 95% CI, 0.31-1.22; *P* = .16). According to subgroup analyses, sex, age, performance status, tumor location, stage, and other pathological factors did not indicate a difference in the prognosis between treatment groups (Figure 3).

### Toxic Effects

Acute toxic effects during or within 6 weeks of treatment completion are listed in Table 2. CRT was well tolerated in both groups. Compared with the capecitabine and oxaliplatin with RT group, the capecitabine with RT group had lower prevalence of anorexia, nausea, vomiting, thrombocytopenia, fatigue, and neuropathy and an increase in the ratio of the level of alanine aminotransferase to bilirubin. Patients who received capecitabine and oxaliplatin with RT had a higher prevalence of grade 3 or 4 toxic effects (ie, nausea, vomiting, and fatigue) than those who received capecitabine with RT. Moreover, there was a significant difference between groups with respect to all grade 3 or 4 toxic effects (114 patients [38.6%] in the capecitabine and oxaliplatin with RT group vs 84 patients [28.6%] in the capecitabine with RT group; *P* = .01).

**Table 2.** Toxic Effects Reported During Treatment or Within 6 Weeks After Treatment Completion

Acute toxic effect	All grades, No. (%)		*P* value	Grade 3-4, No. (%)		*P* value
	Capecitabine with RT	Capecitabine and oxaliplatin with RT		Capecitabine with RT	Capecitabine and oxaliplatin with RT	
Gastrointestinal						
Anorexia	140 (47.6)	183 (62.0)	<.001	1 (0.3)	4 (1.4)	.18
Nausea	88 (30.0)	155 (52.5)	<.001	0	4 (1.4)	.05
Vomiting	28 (9.5)	44 (18.4)	.001	0	4 (1.4)	.05
Diarrhea or proctitis	196 (66.9)	215 (73.1)	.19	60 (20.5)	79 (26.9)	.07
Blood						
Leukopenia	220 (74.8)	210 (71.2)	.78	9 (3.1)	9 (3.1)	.99
Hemoglobin	47 (16.0)	54 (18.3)	.54	0	0	NA
Thrombocytopenia	23 (7.8)	51 (17.3)	.005	0	2 (0.7)	.16
ALT to bilirubin ratio increase	7 (2.4)	18 (6.1)	.05	0	0	NA
Other						
Bodyweight loss	17 (5.8)	19 (6.5)	.94	0	0	NA
Fatigue	163 (55.4)	192 (65.1)	.003	1 (0.3)	8 (2.7)	.02
Radiation dermatitis	192 (65.3)	179 (61.1)	.36	17 (5.8)	9 (3.1)	.11
Neuropathy	6 (2.0)	33 (11.2)	<.001	1 (0.3)	3 (1.0)	.32
Hand–foot syndrome	18 (6.1)	24 (8.1)	.33	0	0	NA
Total	286 (97.3)	290 (98.3)	.40	84 (28.6)	114 (38.6)	.01

Abbreviations: ALT, alanine aminotransferase; NA, not applicable; RT, radiotherapy.

## Discussion

This RCT was conducted to evaluate the efficacy and toxic effects of capecitabine with RT vs capecitabine and oxaliplatin with RT after total mesorectal excision in patients with stage II or III rectal cancer treated primarily with modern RT and standard chemotherapy. Addition of oxaliplatin to capecitabine-based CRT resulted in comparable DFS or OS, but increased severe grade 3 or 4 toxic effects. Consistent with observations in the neoadjuvant setting,^22,23,24,25,26^ this finding provides evidence supporting concurrent capecitabine-based CRT in the adjuvant setting of patients with locally advanced rectal cancer.

Following our phase 1 trials of capecitabine with or without oxaliplatin as postoperative concurrent CRT,^28,29^ this RCT in a Chinese population was crucial to understand the impact of addition of oxaliplatin to capecitabine-based CRT on the efficacy and toxic effects of treatment of patients with stage II or III disease in the adjuvant setting. Consistent with findings from 5 RCTs on neoadjuvant CRT (eTable 2 in Supplement 2),^22,23,24,25,26^ we demonstrated that adjuvant concurrent CRT with capecitabine or capecitabine and oxaliplatin followed by 5-FU or capecitabine and oxaliplatin-based chemotherapy resulted in similar survival outcomes. Several RCTs (ACCORD12,^22^ NSABP R-04,^24^ STAR-01,^25^ and PETACC-6^26^) concluded that 5-FU or capecitabine with or without oxaliplatin concurrent with neoadjuvant RT provided comparable local control and survival for patients with locally advanced rectal cancer. Only 1 RCT (from Germany, CAO/ARO/AIO-04^23^) suggested that neoadjuvant CRT with 5-FU and oxaliplatin resulted in the better pathological complete response and DFS. The 3-year or 5-year DFS in these neoadjuvant RCTs ranged from 62.3% to 75.9%,^22,23,24,25,26^ which was similar to that in our adjuvant RCT. All except 1 RCT^24^ presented a similarly large proportion (ie, approximately 70%) of patients with stage III disease; the 5-year local recurrence of 7.1% with capecitabine with RT and 4.7% with capecitabine and oxaliplatin with RT in our adjuvant RCT was similar to or lower than that in other neoadjuvant trials .^22,23,24,25,26^ Furthermore, 3-year or 5-year OS were comparable in those RCTs, regardless of neoadjuvant or adjuvant CRT (eTable 2 in Supplement 2). Consistently, meta-analyses have indicated that adding oxaliplatin to neoadjuvant concurrent CRT does not improve the prognosis of locally advanced rectal cancer.^30,31^ Our findings and those of other researchers provide evidence of neoadjuvant or adjuvant capecitabine-based CRT in clinical practice.

We demonstrated that adjuvant capecitabine with RT was better tolerated and had a lower prevalence of severe acute toxic effects compared with capecitabine and oxaliplatin with RT. Patients receiving adjuvant capecitabine and oxaliplatin with RT had a lower prevalence of completing concurrent CRT and higher prevalence of toxic effects greater than grade 2 than those receiving capecitabine with RT. Other neoadjuvant RCTs have demonstrated significantly greater severe toxic effects with 5-FU and oxaliplatin–based CRT than those achieved using 5-FU–based single-agent CRT (eTable 2 in Supplement 2).^22,23,24,25,26^ The prevalence of grade 3 or 4 toxic effects was approximately 10% for 5-FU– or capecitabine-based CRT, compared with approximately 25% for 5-FU– or capecitabine and oxaliplatin–based CRT in the neoadjuvant setting. Patients who received postoperative capecitabine-based CRT in our RCT had a higher proportion of grade 3 or 4 toxic effects (28.6%) than that of patients who received single-agent CRT preoperatively (approximately 10%)^22,23,24,25,26^ (eTable 2 in Supplement 2), but this proportion was similar for patients who received preoperative oxaliplatin plus capecitabine or 5-FU–based CRT (approximately 25%) in other RCTs.^22,23,24,25,26^ However, in the setting of postoperative CRT, the prevalence of grade 3 or 4 toxic effects observed with capecitabine with RT in our RCT was lower than that with single-agent CRT from other comparative RCTs (40%-50%).^10,11,12^ The reduced prevalence of severe toxic effects with adjuvant capecitabine with RT in our RCT was probably owing to the homogeneity and reduced dose to healthy tissues thanks to use of IMRT. The significantly different toxicity profile between single-agent and dual-agent CRT also supports use of 5-FU– or capecitabine-based CRT in neoadjuvant or adjuvant settings.

Our study had 4 main strengths. First, we used contemporary RT strategies (IMRT and 3D-CRT). Second, we used contemporary adjuvant chemotherapy regimens. Third, our focus was on patients who received postoperative CRT. Neoadjuvant RCTs comparing the efficacy and toxic effects of the addition of oxaliplatin to capecitabine or 5-FU CRT have usually used conventional radiation methods (ie, 3 or 4 box fields),^22,23,24,25,26^ and variably allowed delivery of a tumor-bed boost^24^ and adjuvant chemotherapy regimens.^22,23,26^ Almost all our patients were treated with IMRT or 3D-CRT. Fourth, to our knowledge, this was the first RCT to compare capecitabine with RT with capecitabine and oxaliplatin with RT in the adjuvant setting for patients in China with stage II or III rectal cancer. Despite use of adjuvant CRT for such patients, local control and survival were favorable in treatment groups, probably because of homogeneous dose distribution with IMRT and 3D-CRT and use of contemporary adjuvant chemotherapy regimens.^32^ The favorable oncologic outcomes in this multicenter RCT indicate the feasibility of routine use of IMRT for rectal cancer.

### Limitations

Our study has some limitations. First, we underestimated DFS in the control group, which led to a failure to reach the expected number of events. Further extension of the follow-up period may lead to more events, but we believe that the increase in the number of DFS events will be very limited after 5 years of follow-up. Second, our RCT was restricted to patients who received postoperative concurrent CRT. Given a high prevalence of severe toxic effects and a higher risk of recurrence with postoperative capecitabine with RT,^10,11,12^ use of neoadjuvant CRT as a standard of care has increased in the 21st century (especially in Western countries). However, the survival and local control outcomes in our RCT compared favorably with those in other neoadjuvant RCTs.^22,23,24,25,26^ Hence, capecitabine with RT could be considered to be an alternative type of multidisciplinary management of locally advanced rectal cancer for patients who did not receive neoadjuvant CRT, particularly in China.

## Conclusions

This RCT found that addition of oxaliplatin to capecitabine-based postoperative CRT did not improve the efficacy of treatment but increased the risk of severe acute toxic effects. These findings highlights the basic role of postoperative capecitabine with RT for patients with locally advanced rectal cancer.

## Notes

Supplement 1.
Trial Protocol and Statistical Analysis Plan

Supplement 2. **eTable 1.** Completion of Adjuvant Chemoradiotherapy and Chemotherapy **eTable 2.** Overview of Randomized Clinical Trials of Adding Oxaliplatin to 5-FU– or Capecitabine-Based Chemoradiotherapy for Rectal Cancer in Neoadjuvant or Adjuvant Settings

Supplement 3.
Data Sharing Statement
