# Supplementary material for: Postoperative Chemoradiotherapy With Capecitabine and Oxaliplatin vs Capecitabine for Stage II to III Rectal Cancer: A Randomized Clinical Trial
Source: JAMA Netw Open. 2021 Nov 30;4(11):e2136116. doi: 10.1001/jamanetworkopen.2021.36116 (PMC8634060; doi:10.1001/jamanetworkopen.2021.36116)
Supplement: Supplement 1. — Trial Protocol and Statistical Analysis Plan [file jamanetwopen-e2136116-s001.pdf]

Postoperative concurrent chemoradiotherapy with or without Oxaliplatin in pathological stage II and III rectal cancer: A randomized multicenter phase III trail

Trail Code：NCT00714077(ClinicalTrials.gov)

Version：1.0

Date：08-03-15

Principle center：Department of Radiation Oncology,

Chinese Academy of Medical Sciences

Principal Investigator：Ye-Xiong Li

Tel： 87788860(O), 13801103877，yexiong3@yahoo.com.cn

Research design

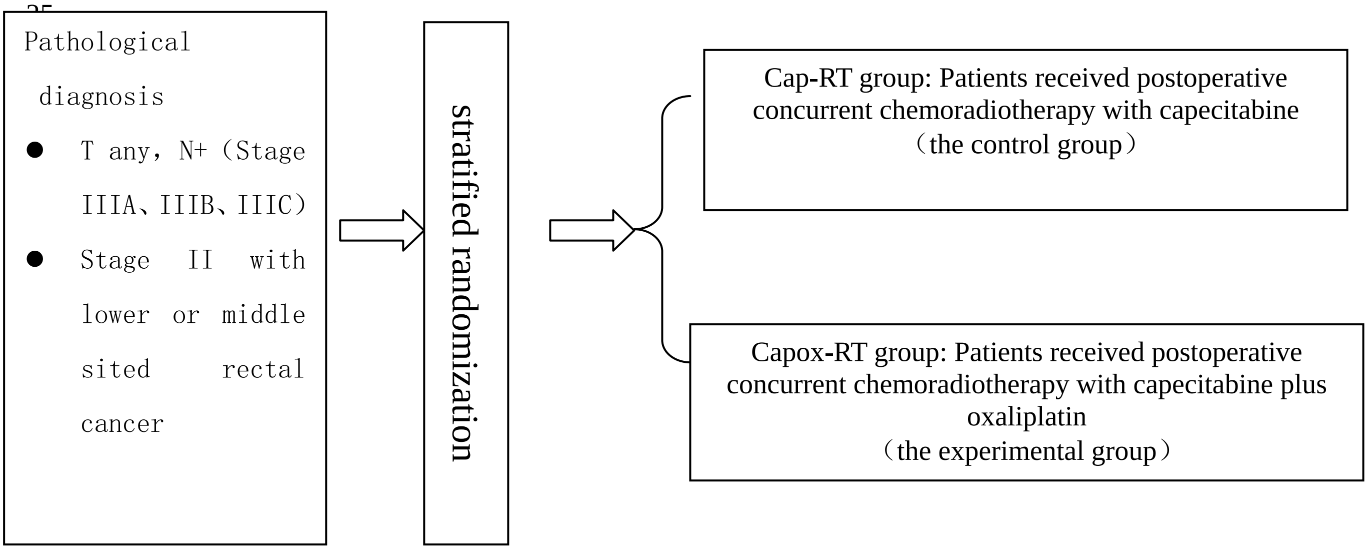

Contents

Research outline 02/26

1. Introduction 04/26
2. Objectives 07/26
3. Methods and steps 08/26
4. Statistics 14/26
5. Follow up 14/26
6. Adverse Drug Reactions and Management 14/26
7. The principle of dosage modification 15/26
8. Adverse drug reaction reporting system 15/26
9. Serious adverse reaction reporting system 15/26
10. Management principles 16/26
11. Lists and contact details for multiple centers 16/26
12. Research Progress 16/26
13. Patient informed consent 18/26
14. Appendix
    - 14.1 Karnofsky scoring criteria 20/26
    - 14.2 Common Terminology Criteria for Adverse Events（CTC 3.0）(part) 22/26
    - 14..3 Clinical staging of rectal cancer（UICC/AJCC，2002） 24/26
    - 14.4 Quality of life scale（EORCT QOL C30） 25/26

1 Introduction

Colorectal adenocarcinoma is the third most common malignancies in the United States and the third most common cause of cancer-related death. In China, it accounts for the fourth most common malignancy. With the improvement of people's living standards in our country, the incidence of colorectal cancer is increasing, and it has become a major disease that threatens the health of people. Unlike the distribution of colorectal cancer abroad, China's rectal cancer accounts for about half of all colorectal cancer. Although surgery is the treatment of choice, the local recurrence rate was 15%-65%, especially for stage II and III rectal cancer patients. This severely affects the long-term survival of patients 5-Fluorouracil (5-FU)-based concurrent chemoradiotherapy as an adjuvant approach has resulted in reduced local recurrence and improved survival in patients with Stage II or III rectal cancer. Adjuvant concurrent chemoradiotherapy are considered the standard of care in the clinical practice for stage II or III resected rectal cancer. However, studies indicated that local recurrence was 15-20% and distant metastasis remains as great as 30-40% for patients who received capecitabine based postoperative chemoradiotherapy. New cytotoxic agents or new combinations of chemotherapeutic drugs have been investigated to reduce the rate of distant failure in patients with resected rectal cancer. Studies demonstrated that OXA or Cap based regimen significantly improved compared with 5-FU based chemotherapy in patients with high risk colon cancer after curative surgery

Oxaliplatin is a third-generation platinum derivative with the same cytotoxic effect as cisplatin, but oxaliplatin overcomes the toxicity of cisplatin to the gastrointestinal tract and kidneys. The randomized trials have proved that the FOLFOX4 scheme, which is mainly based on oxaliplatin, is superior to the previous 5-FU / LV scheme, whether it's chemotherapy for advanced colorectal cancer or adjuvant chemotherapy for colon cancer. Therefore, FOLFOX4 has replaced the classic 5-FU / LV regimen as the standard chemotherapy regimen for colorectal cancer.

Capecitabine, a member of the new class of oral fluoropyrimidines, was designed to mimic continuous 5-FU infusion. As an oral agent, capecitabine is more convenient than 5Fu. The results demonstrated that capecitabine yielded as effective as intravenous 5-FU plus leucovorin, and has a favorable safety profile.

Both Oxa and capecitabine has radio sensitization properties in vitro. The reported toxicity profile was similar to that of our previous studies, In our previous Phase I trial, we evaluated CAP alone with concurrent RT in postoperative treatment of rectal cancer. We found that the recommended dose of capecitabine was 1600 mg/m^2/d administered from days 1-14 with a 7 days rest for two cycles. The subsequent phase II prospective clinical trial confirmed the better tolerability and safety of capecitabine concurrent chemoradiotherapy. Last year, a phase I clinical study of oxaliplatin combined with capecitabine was further completed. The trial indicates that the maximum tolerable dose of oxaliplatin combined with 1300 mg/m^2/d was 70 mg/m^{2}per week. In prospective clinical phase II studies show that more grade 3-4 toxicities was observed in the oxaliplatin combined with capecitabine group than in the capecitabin group. Inclusion of oxaliplatin in the capecitabine based postoperative chemoradiotherapy was tolerable and feasible. Based on these results, we designed a prospective, randomized, phased III trail to see if oxaliplatin incorporated capecitabine concurrent chemoradiotherapy in postoperative setting could improve survival compared with capecitabine alone.

In the clinical setting, there are few predictive factors of response to the treatment in patients with

colorectal cancer. Some researches show that the over expression of Epidermal growth factor receptor (EGFR), vascular endothelial growth factor (VEGF) and BCL-2, and the mutated p53 gene have prognostic significance in patients with colorectal cancer. Polymorphisms of thymidylate synthetase (TS) and DPD enzyme genes, expression status of DNA repair genes, etc. have been associated with the response to adjuvant treatment in locally advanced rectal cancer. Survivin、c-erbB-2、gammaH2AX、Ku80, etc. are related to radio sensitivity. However, the potential clinical use of these bio-markers needs further evaluation in prospective clinical trials. Although it is clear from the literature that colorectal cancer may be regulated by many different genes. Gene can affect radiotherapy or chemotherapy sensitivity. The reason may include two aspects. (1)Differences in each individual's innate genetic qualities. For example, many studies demonstrate that different individuals have different metabolism and detoxification capabilities for specific drugs, this cause different patients to respond differently to the same dose of chemotherapy. Both radiation and chemotherapy will induce apoptosis in neoplastic cells through DNA damage, and there are complex DNA repair mechanisms with significant differences among individuals. DNA repair ability may play an important factor in determining chemoradio-sensitivity. (2)There are significant differences in gene mutation or tumor cell expression in different patients. For example, some patients with colorectal cancer have mutations in tumor suppressor genes such as P53 and APC or overexpression of EGFR and VEGF, while others has no. These changes in gene and protein expression profiles between individuals have been suggested as indicators of tumor metastasis or recurrence. Therefore, systematic and comprehensive research is necessary to explore the treatment sensitivity and prognosis of colorectal cancer. Then these markers could be validated for selecting the optimal personalized treatment in individual patients. This is also the current research hotspot in the field of international oncology and an important scientific problem that needs urgent breakthrough.

The development of gene chip and tissue chip technology in recent years has provided us with a high-throughput method for screening related candidate genes. For example, the expression chip can quickly detect the expression profiles of a large number of genes; SNP chip can scan 500,000 SNPs across the genome; Tissue chips allow high-throughput immunohistochemical analysis. These developments have provided technical support for our research. A recent German study showed that 23 patients with rectal cancer received concurrent neoadjuvant radiotherapy with 5Fu regimen. All patients underwent surgery. The end point was the degree of postoperative pathological reaction. 54 genes were significantly up-regulated or down-regulated. These gene changes reflect the sensitivity of 5-FU and concurrent radiotherapy, but the relationship with long-term efficacy is under study.

This project is a prospective phase III randomized study. Based on the postoperative pathological results, patients with stage II / III rectal cancer after radical mastectomy are selected, and concurrent chemoradiotherapy and effect evaluation are performed according to the established protocol. Biopsy specimens are collected during surgery for cryopreservation, and 3-5ml of peripheral blood is collected from patients before concurrent chemoradiotherapy at convenient. Combined with the treatment efficacy, the gene profile and single nucleotide polymorphisms related to chemoradiotherapy sensitivity were analyzed. Results from previous studies suggest that our candidate genes may play a major role in screening genetic variation, and that corresponding genes may lead to significant difference at the results in chemo radiotherapy. The

purpose of this study was to find genetic variants of genes that are significantly associated with chemosensitivity. Correlation analysis of gene samples and SNPs that were significantly related to chemoradiotherapy sensitivity was screened to further verify the relationship with radiochemotherapy sensitivity. At the same time, the biological function of these gene profiles and SNPs was studied.

In conclusion, this research is based on standardized clinical treatments and is closely integrated with basic research. Through the prospective phase III randomized study, a better concurrent chemoradiotherapy protocol was obtained, which improve overall survival rate and reduce local regional recurrence rate.

References

1. Tveit KM, Guldvog I, Hagen S, et al: Randomized controlled trial of postoperative radiotherapy and short-term time-scheduled 5-fluorouracil against surgery alone in the treatment of Dukes B and C rectal cancer. Norwegian Adjuvant Rectal Cancer Project Group. Br J Surg 84:1130-5, 1997
2. Wolmark N, Wieand HS, Hyams DM, et al: Randomized trial of postoperative adjuvant chemotherapy with or without radiotherapy for carcinoma of the rectum: National Surgical Adjuvant Breast and Bowel Project Protocol R-02. J Natl Cancer Inst 92:388-96, 2000
3. Prolongation of the disease-free interval in surgically treated rectal carcinoma. Gastrointestinal Tumor Study Group. N Engl J Med 312:1465-72, 1985
4. Krook JE, Moertel CG, Gunderson LL, et al: Effective surgical adjuvant therapy for high-risk rectal carcinoma. N Engl J Med 324:709-15, 1991
5. de Gramont A, Figer A, Seymour M, et al: Leucovorin and fluorouracil with or without oxaliplatin as first-line treatment in advanced colorectal cancer. J Clin Oncol 18:2938-47, 2000
6. Giacchetti S, Perpoint B, Zidani R, et al: Phase III multicenter randomized trial of oxaliplatin added to chronomodulated fluorouracil-leucovorin as first-line treatment of metastatic colorectal cancer. J Clin Oncol 18:136-47, 2000
7. Andre T, Boni C, Mounedji-Boudiaf L, et al: Oxaliplatin, fluorouracil, and leucovorin as adjuvant treatment for colon cancer. N Engl J Med 350:2343-51, 2004
8. Van Cutsem E, Hoff PM, Harper P, et al: Oral capecitabine vs intravenous 5-fluorouracil and leucovorin: integrated efficacy data and novel analyses from two large, randomised, phase III trials. Br J Cancer 90:1190-7, 2004
9. Twelves C, Wong A, Nowacki MP, et al: Capecitabine as adjuvant treatment for stage III colon cancer. N Engl J Med 352:2696-704, 2005
10. Sawada N, Ishikawa T, Sekiguchi F, et al: X-ray irradiation induces thymidine phosphorylase and enhances the efficacy of capecitabine (Xeloda) in human cancer xenografts. Clin Cancer Res 5:2948-53, 1999
11. Cividalli A, Ceciarelli F, Livdi E, et al: Radiosensitization by oxaliplatin in a mouse adenocarcinoma: influence of treatment schedule. Int J Radiat Oncol Biol Phys 52:1092-8, 2002
12. Kim JC, Kim TW, Kim JH, et al: Preoperative concurrent radiotherapy with capecitabine before total

mesorectal excision in locally advanced rectal cancer. Int J Radiat Oncol Biol Phys 63:346-53, 2005

13. Kuebler JP, Wieand HS, O'Connell MJ, et al: Oxaliplatin combined with weekly bolus fluorouracil and leucovorin as surgical adjuvant chemotherapy for stage II and III colon cancer: results from NSABP C-07. J Clin Oncol 25:2198-204, 2007
14. Jin J, Li YX, Liu YP, et al: A phase I study of concurrent radiotherapy and capecitabine as adjuvant treatment for operable rectal cancer. Int J Radiat Oncol Biol Phys 64:725-9, 2006
15. Souglakos J, Androulakis N, Mavroudis D, et al: Multicenter dose-finding study of concurrent capecitabine and radiotherapy as adjuvant treatment for operable rectal cancer. Int J Radiat Oncol Biol Phys 56:1284-7, 2003
16. Ngan SY, Michael M, Mackay J, et al: A phase I trial of preoperative radiotherapy and capecitabine for locally advanced, potentially resectable rectal cancer. Br J Cancer 91:1019-24, 2004
17. Dunst J, Reese T, Sutter T, et al: Phase I trial evaluating the concurrent combination of radiotherapy and capecitabine in rectal cancer. J Clin Oncol 20:3983-91, 2002
18. Rodel C, Grabenbauer GG, Papadopoulos T, et al: Phase I/II trial of capecitabine, oxaliplatin, and radiation for rectal cancer. J Clin Oncol 21:3098-104, 2003
19. Fakih MG, Rajput A, Yang GY, et al: A Phase I study of weekly intravenous oxaliplatin in combination with oral daily capecitabine and radiation therapy in the neoadjuvant treatment of rectal adenocarcinoma. Int J Radiat Oncol Biol Phys 65:1462-70, 2006
20. Glynne-Jones R, Sebag-Montefiore D, Maughan TS, et al: A phase I dose escalation study of continuous oral capecitabine in combination with oxaliplatin and pelvic radiation (XELOX-RT) in patients with locally advanced rectal cancer. Ann Oncol 17:50-6, 2006
21. Machiels JP, Duck L, Honhon B, et al: Phase II study of preoperative oxaliplatin, capecitabine and external beam radiotherapy in patients with rectal cancer: the RadiOxCape study. Ann Oncol 16:1898-905, 2005
22. Schmoll HJ, Cartwright T, Tabernero J, et al: Phase III trial of capecitabine plus oxaliplatin as adjuvant therapy for stage III colon cancer: a planned safety analysis in 1,864 patients. J Clin Oncol 25:102-9, 2007
23. De Paoli A, Chiara S, Luppi G, et al: Capecitabine in combination with p reoperative radiation therapy in locally advanced, resectable, rectal cancer: a multicentric phase II study. Ann Oncol 17:246-51, 2006
24. Jing Jin, Yexiong Li, Weihu Wang, et al. Phase I study of oxaliplatin in combination with capecitabine and radiotherapy as postoperative treatment for stage II and III rectal cancer. In press
25. Rodel F, Hoffmann J, Grabenbauer GG, *et al.* High survivin expression is associated with reduced apoptosis in rectal cancer and may predict disease-free survival after preoperative radiochemotherapy and surgical resection. Strahlenther Onkol 178:426-35, 2002
26. Ruzzo A, Graziano F, Loupakis F,et al. Pharmacogenetic profiling in patients with advanced colorectal cancer treated with first-line FOLFOX-4 chemotherapy. J Clin Oncol 25:1247-54, 2007
27. Gordon MA, Gil J, Lu B, et al. Genomic profiling associated with recurrence in patients with rectal cancer treated with chemoradiation. Pharmacogenomics 7:67-88, 2006
28. Stoehlmacher J, Park DJ, Zhang W,et al. A multivariate analysis of genomic polymorphisms: prediction of

clinical outcome to 5-FU/oxaliplatin combination chemotherapy in refractory colorectal cancer. Br J Cancer 91:344-54, 2004

2 Objectives

2.1、a randomized phase III trail of postoperative concurrent capecitabine and radiotherapy with or without oxaliplatin for pathological stage II/III rectal cancer

2.1.1 to evaluate the result of postoperative concurrent capecitabine and radiotherapy with or without oxaliplatin

a) 3-year local recurrence rate (3y LR)
b) 3-year disease-free survival rate 3 (3y DFS)
c) 3-year overall survival rate (3y OS)
d) 3-year distant metastasis rate (3y DM)

2.1.2 to evaluate the grade 3-4 toxicities in the two different groups

2.2、Quality of life assessment

2.3.1 Evaluate the quality of life of patients receiving concurrent chemoradiotherapy with or without Oxaliplatin.

3 Methods and steps

3.1. Prospective phase III clinical randomized study

3.1.1. Eligibility criteria

1) Sign informed consent
2) 18-75 years old。
3) Had a R0 total mesorectal excision with adenocarcinoma
4) Pathological diagnosis
   - T any，N+（pathological stage III）
   - Stage II with lower or middle sited rectal cancer（The tumor center is below the peritoneal reflex line）
5) The interval between the date of surgery and the date of enrollment≥2 weeks, ≤3 months
6) The upper border of the tumor was below L5。
7) Karnofsky performance status≥70；Life expectancy >6 month.
8) Before enrollment, chemotherapy cycle was less than 4 cycles. The interval between the start of radiotherapy and the operation time should not exceed six months
9) No previous pelvic radiation therapy
10) hemoglobin≥100 g/L，leucocytes≥3.5×109 /L，neutrophils≥1.5X109, platelets≥100×109 /L。CR≤1.5 x the upper limit of normal(ULN)，TB≤2.5 x ULN，AST&ALT≤2.5 x ULN，AKP≤2.5 x ULN。
11) No peripheral neuropathy

3.1.2.Exclusion criteria

1) Patients who suffered from previous malignancies, except adequately treated non-melanin pathology skin cancer, and *in situ* cervical cancer.
2) Patients have colon cancer, whose stage ≥II
3) Patients who are pregnant or lactating
4) Patients who are in child-bearing age, and do not contraception
5) Hypersensitivity reactions related to Platinum and fluorouracil drugs
6) patients with peripheral nervous system disorders (Whether it is a secondary or primary disease)
7) Patients suffering from some severe disease. For example: Large-scale myocardial infarction, cardiac function ≥ Grade II, history of mental illness, and severe diabetes.
8) Patients had participated in another clinical trial within 4 weeks before the start of treatment.
9) Patients concurrently treated with other anticancer drugs
10) Patients have a history of organ transplant

3.1.3. Criteria for Exit from the Treatment Protocol

- Patients suffering from distant metastasis
- Patients withdraw from the study at any point for ang reason。

3.1.4. Criteria for Removal from the Treatment Protocol

- Patient violates study protocol requirements
- Incomplete, inaccurate, and poor quality of data records
- The patient refused treatment due to non-medical factors.

3.1.5.：Research steps

3.1.5.1.surgery：

- treatment was started no longer than 3 months after surgery；
- Radical resection (R0), specific surgical methods are not required.
- TME surgery is recommended
- Collect living tissue during surgery and send it for cryopreservation, preparing for further basic research

3.1.5.2.Pre-clinical examination

- The pretreatment evaluation had to be performed within 4 weeks before study entry:
  - ✧ Magnetic resonance imaging (MRI) or enhanced computed tomography (CT) of the pelvic region；
  - ✧ Ultrasound or CT of the abdomen；
  - ✧ Chest x-ray。
- The pretreatment evaluation had to be performed within 2 weeks before study entry:
  - ✧ Medical history；
  - ✧ KPS；
  - ✧ Height, weight and vital signs；
  - ✧ Physical examination and Oncology examination；

- ✧ Complete blood cell counts, liver and renal function test；
- ✧ CEA、CA199；

3.1.5.3.Radiation Therapy

- Radiation dose： 50Gy in 25 fractions of 2.0Gy, five times per week, over 5 weeks。
- Radiation method：adopt three field technique or Intensity-modulated radiotherapy (IMRT) technique
  - ✧ A three field technique：
    - ✓ Position: For patients undergone Dixon surgery, inject about 20-50ml barium through the anus before position, or put a metal mark on the original anus and the perineal scar. Patients use belly-board (recommended) in the prone position. A posterior lateral field and a lateral field were irradiated at a dose ratio of 2: 1: 1, and the lateral field was a 30-degree wedge plate.
    - ✓ Target Definition: included the tumor bed, the soft tissue before sacrum, perineal scar and internal iliac lymphatic drainage area. The upper border of the filed corresponded to the lower edge of L5. The lower border should more than 1cm below the lower edge of the perineal scar (Mile’s), or be the lower margin of obturator (Dixon operation). The lateral border was 1cm outside of the true pelvis. The posterior border of the bilateral field includes the lateral patella cortex. The front border includes 2-3 cm range before the anterior wall of the rectum (Dixon surgery), or the posterior 1/3 of the bladder depending on postoperative pelvic CT scans(Mile’s) (Figure 1)..

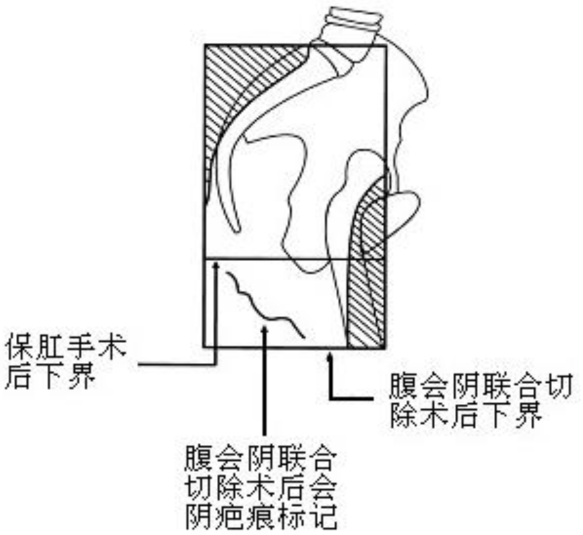

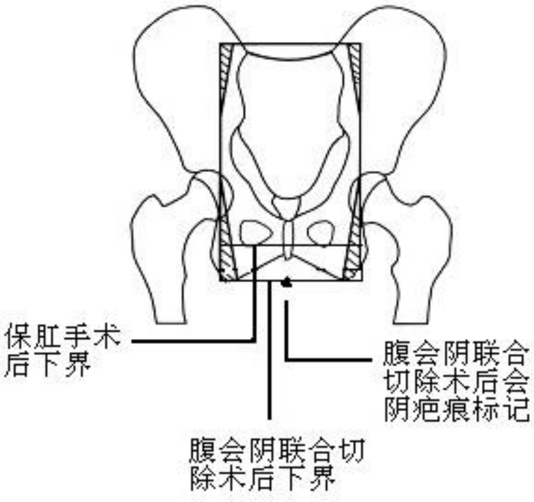

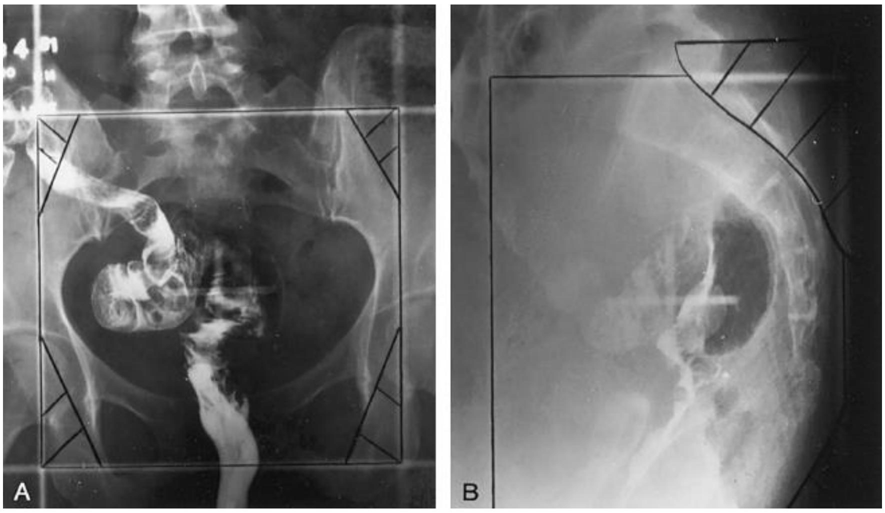

Fig 1：show the three field technique

- ✧ IMRT：
  - ✓ Preparation before position: The patient emptied the bladder 1.5 hours before positioning, Oral mirage glucosamine 20ml + 1000-1500ml water is taken orally at intervals of about half an hour, 400 ~ 600ml each time, and urine is held at the same time.
  - ✓ Position and Fixed Position：in the prone position, use belly-board (recommended), and immobilization in a thermo plastic body film mold
  - ✓ CT simulation: All patients underwent CT-based treatment planning with intravenous contrast. CT was scanned at a layer thickness of 0.5 cm, and about 50 to 80 CT images were collected. However, if the patient is allergic to the contrast agent or is old, or has comorbidities, he could perform a plain scan. The patient drank the same volume of water 1.5 hours before treatment. The degree of bladder filling during treatment is similar to the position state.
  - ✓ Target Definition:

- clinical tumor volume (CTV): The clinical tumor volume (CTV) included the mesorectum, presacral, anastomosis, outer iliac vessels and part of common iliac vessels above the sacrum 3, and internal iliac lymphatic drainage area. Perineal scar was included in patients who undergo Mile 's surgery. Sciatic rectum fossa was included in those with disease locating in the lower or middle rectum.
- Specific details of the target volume: The upper border of the filed corresponded to the lower edge of L5. The mesorectum regions was included in patients with cancer locating in the upper of the rectum. The lower boundary should more than 3cm below the lower edge of the anastomosis, and it may not include all of the sciatic rectal fossa; The mesorectum regions, the sciatic rectal fossa and the perineal scar were included in patients with cancer locating in the lower or middle of the rectum. The lateral border was the inner edge of the true pelvis, the front border includes the 1/4 ~ 1/3 of the posterior wall of the filling bladder, The posterior border included half of the sacral cortex (above the upper edge of iliac crest 3) and the posterior margin of the sacral cortex (below the upper edge of iliac crest 3) (Figure).
- planning target volume (PTV): The planning target volume (PTV) was a 10-mm expansion of the CTV at the head and foot direction, a 5-10-mm expansion at the left and right direction, a 5-10-mm expansion at the up and down direction, .
- Contour of the normal tissues: The normal tissues include the residual rectum, bilateral femoral head, bladder, the small intestine which needs to be delineated to the top 10 layers of PTV, and testes.
- Dosimetric evaluation: The minimum dose that 95% PTV receives is DT50Gy. The maximum dose within the irradiation range is ≤58Gy, and the maximum dose area must not be located on the small intestine or the residual rectum. The minimum dose of PTV should not be lower than 46.5Gy.
- Evaluation of normal tissue Dose-Volume Histogram
  - Femoral head：D5≤50Gy
  - Bladder：D50≤50Gy
  - Residual rectum：Dmax≤53Gy
  - Small intestine：D50≤20-30Gy，Dmax≤50Gy
  - Testes：evaluate the Dmax and median dose of testes.

3.1.5.4. Concurrent chemotherapy regimen

- Cap-RT group （the control group）：radiotherapy with concurrent oral capecitabine (1600mg/m^2/d) on days 1-14 and 22-35. Cap was administered in whole tablets based on body surface area.
- Capox-RT group（the experimental group）：radiotherapy with concurrent oral capecitabine

(1300mg/m^2/d) on days 1-14 and 22-35 and a 2-h infusion oxaliplatin (60mg/m^2/week) on weeks 1,2,4 and 5. Oxaliplatin was administered at the actual dose calculated from body surface area (loxadine @ was recommended). Add oxaliplatin to 250-500 ml of 5% glucose solution for intravenous infusion for a duration of 2-6 hours, the intravenous infusion time was not less than 2 hours. Do not use with alkaline drugs or sodium chloride solution, do not mix with other drugs, avoid cold irritation when infusing oxaliplatin.

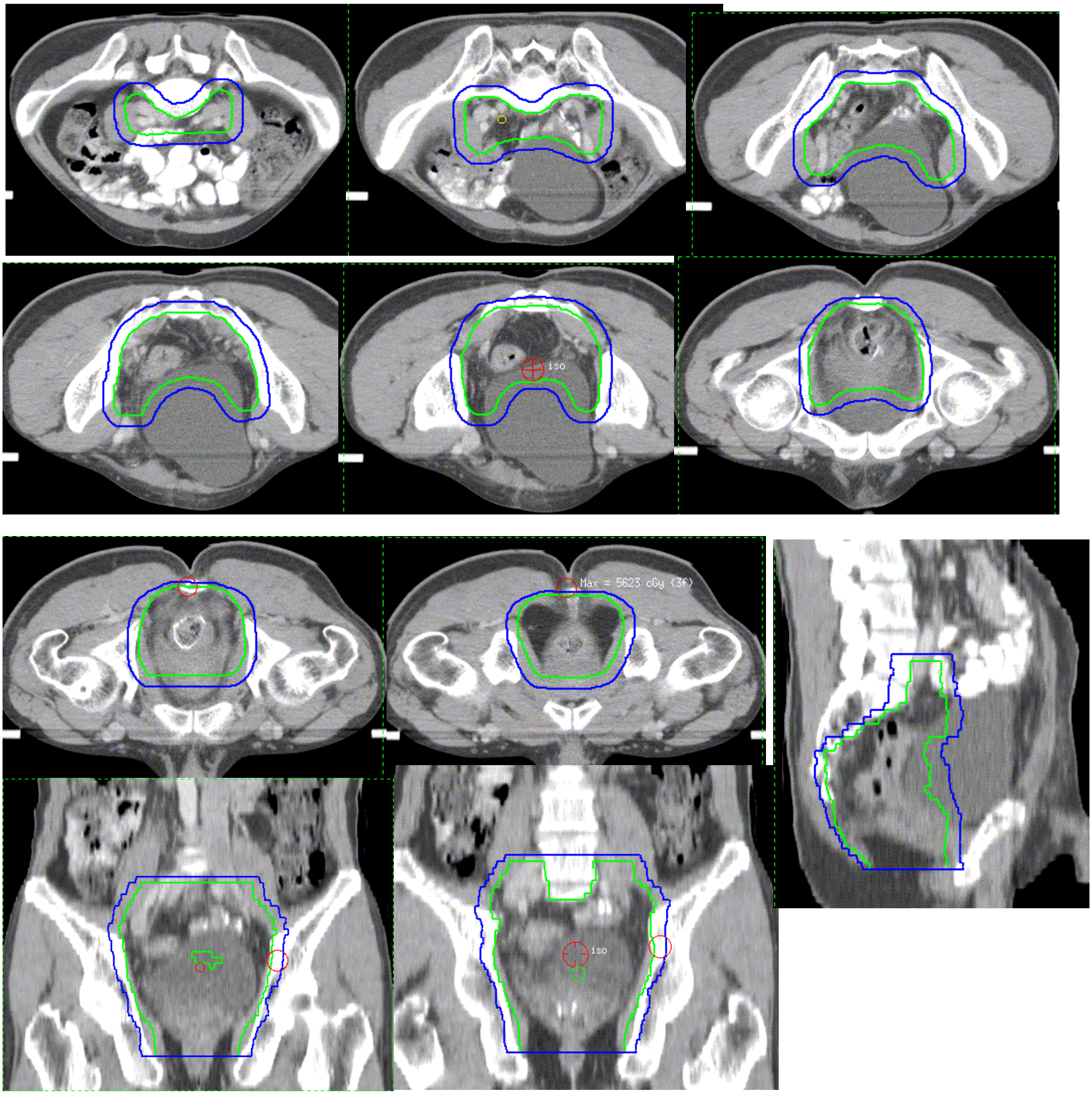

Contour of CTV/PTV

3.1.5.5. Adjuvant chemotherapy： After chemoradiotherapy is completed, 4~6 cycles of xelox or 8~12 cycles of FOLFOX was deliverd.

3.1.5.6. Technical route:

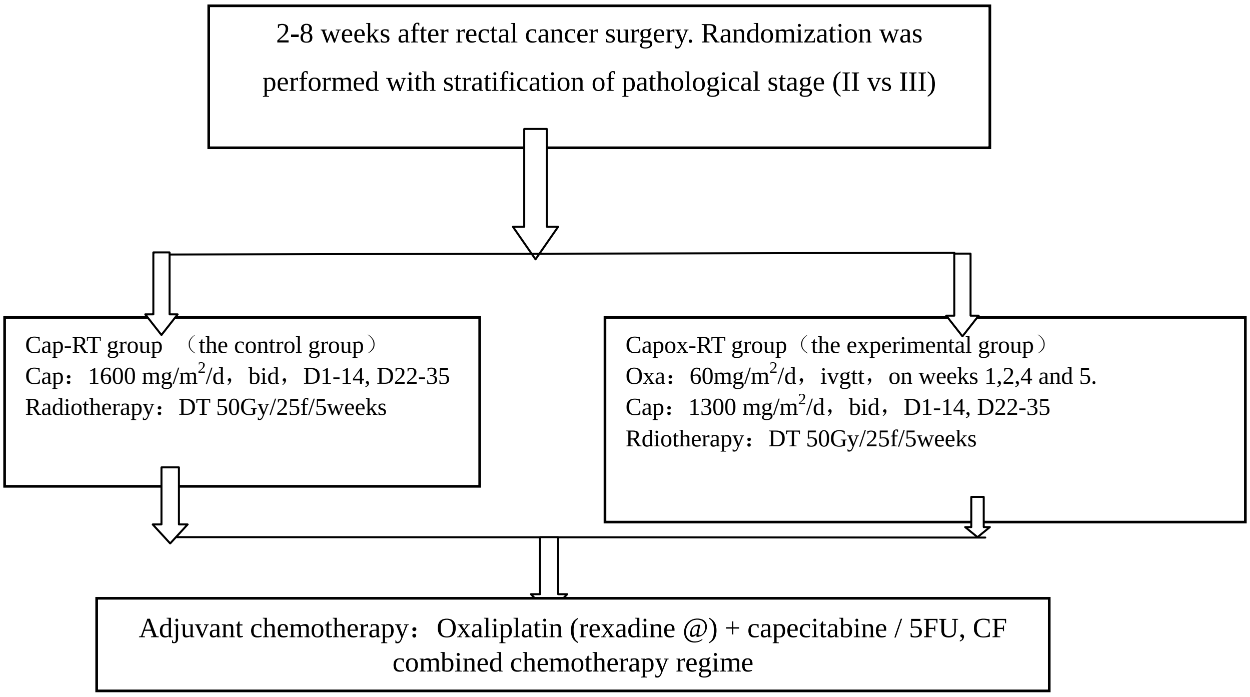

3.2. Quality of life assessment

3.2.1 objective: Stage II / III rectal cancer patients participating in a prospective phase III randomized clinical trial of capecitabine, with or without oxaliplatin concurrent radiotherapy

3.2.2. Method

a) Quality of life were monitored before the treatment, weekly during chemoradiotherapy and after each adjuvant chemotherapy cycle and semi-annually in the first year after treatment
b) The evaluation form is EORTC QOL C30 scale
c) Comparing the quality of life of patients receiving different concurrent chemoradiotherapy regimens

4 Statistics

4.1. The clinical part:

a) Sample size calculation:

This is a multicenter, randomized, phase III trail. Statistical tests were based on a two-sided significance level. A p value of <0.05 was considered statistically significant. The primary endpoint was 3y-DFS. Assuming a 10% dropout rate, a total of 570 patients (285 per group) provided 80% power to detect 3-year DFS of 65% increased to 75%, with a two-sided α of 0.05. The duration of treatment and follow-up was three years.

b) Statistical methods:

The outcomes were evaluated in the intention-to-treat analysis and the per-protocal analysis. Statistical analysis was carried out using SPSS or SAS. Count data are described by rates and percentages, and measurement data are described by means, standard deviations, and 95% confidence intervals. DFS

and OS were estimated with Kaplan-Meier method and the log Rank test. chi-square and mann-whitney u tests were used to compare the differences of categorical viriables and continuous variables. Cox proportional hazards model was used for prognostic analysis. Statistical inferences were made using probability P values, and the statistically significant cutoffs were expressed as 0.05.

4.2 The Lab part:

Multivariate unconditional logistic regression model were used to compare the difference clinical results of the different genotypes. The odds ratio (OR) and its 95% confidence interval (CI) should be calculated. Statistical tests were based on a two-sided significance level. A p value of <0.05 was considered statistically significant. Statistical analysis was carried out using Statistical Analysis System（SAS, 6.12v）

4.3 Quality of life

Calculate the score of each evaluation form according to the EORTC life list, the t test was used to compare the differences.

5 Follow up

- Patient follow up should be performed at least every 3 months during the first two years. The follow-up content includes physical condition score, physical examination, advanced treatment response, CEA and other tumor markers and quality of life assessment; thoracotholateral radiograph (once every six months), abdominal ultrasound / CT (once every six months), and pelvic CT (Once every six months), colorectal once a year.
- Then the follow up should performed at least every 6 months during 2-5 years after treatment. The follow-up content includes physical condition score, physical examination, advanced treatment response, CEA and other tumor markers; thoracic and lateral radiography, abdominal ultrasound / CT, and quality of life assessment
- Deadline of the follow-up: 3 years after the completion of all treatments.

6 Management of adverse reactions:

The acute toxicity was scored according to the Common Terminology Criteria for Adverse Events (CTCAE 3.0/RTOG)，Examination and grading for subjective and objective evidence of toxicity in patients will be carried out in case report form (CRF). Serious adverse reactions should be reported to the hospital ethics committee in writing within 24 hours. At the same time, patients would be treated promptly. All patients with severe adverse reactions should be followed up to recovery.

- Nausea, vomiting: Metoclopramide, domperidone, or H-3 receptor blockers can be given.
- Diarrhea, gastroenteritiss：Oral Smecta and Yimengting. If dehydration occurs, infusion and anti-inflammatory treatment can be given according to specific conditions.
- Hematological side effects: Oral blood-raising drugs or subcutaneous injections of colony-stimulating factors, erythropoietin, or platelet-producing drugs
- Hand-foot syndrome：Neurotrophic medications, such as Vit B1, B12, etc.
- Skin side effects：Topical treatments such as hydrogen oil, Biafine, and Jinin peptide.

- Hypersensitivity reaction：Give anti-allergic treatment.
- Fever: physical cooling, symptomatic antipyretic treatment, or anti-inflammatory treatment.
- Management of postoperative complications: If obstruction, anastomotic leakage / stenosis or wound does not heal after surgery, it should be recorded first, then respond positively according to surgical procedures.

7 The principle of dosage modification

7.1. Dose reductions for toxicity

- Treatment was continued without modification for Grade 1 toxicity. All can treated symptomatically.
- If a patient experienced any more than Grade 3 non-hematological toxicity, or ≥G3 anemia and thrombocytopenia, ≥G4 hematological toxicity, the dose of treatment should be reduced. In CapOx group, the dose of Oxaliplatin was reduced to 20% of the original dose(60 mg/m^2/week reduced to 48 mg/m^2/week）, while capecitabine was continued；In Cap group, the dose of capecitabine was reduced to 20% of the original dose（1600 mg/m^2/week reduced to 1280 mg/m^2/week）. When the severity had decreased or did not get worse, keep the reduced dose. In principle, the original dose would not be used.
- If a patient's adverse reactions worsen, or If a patient experienced any more than Grade 3 non-hematological toxicity, or ≥G3 anemia and thrombocytopenia, ≥G4 hematological toxicity again, We should stop chemotherapy. For short, oxaliplatin only received one reduction.

7.2 Principles of Stop / Delay Radiotherapy

- When occur acute intestinal obstruction (incomplete or complete obstruction), stop chemoradiotherapy. Obstructive remission with conservative treatment can continue to chemoradiotherapy.
- When KPS score is less than 50 points, stop chemoradiotherapy. Chemoradiotherapy can be continued when symptomatic treatment is relieved.
- If a patient experienced Grade 4 toxicity, When the severity get worse, stop chemoradiotherapy.

8 Adverse Drug Reactions (ADRs) reporting system

During the treatment period, patients should be carefully observed, and degrees of adverse drug reactions during the treatment should be evaluated according to CTC 3.0 and recorded in the CRF table. It should be reported to the GCP center of the clinical research sponsor and the main person in charge of the research project within 3 days, if the following serious adverse drug reactions occur.

- Any fatal adverse drug reaction.
- Any deaths related to the drugs in this study
- Adverse drug reactions significantly worsened in this study
- The occurrence of acute myelogenous leukemia: the time of occurrence, diagnosis, and time interval from the use of the drug in this study should be reported.

9 Serious Adverse Effects (SAEs) reporting system

8.1 Definition of SAEs： Any serious adverse events that occurred during treatment, whether related to this study or not, should be reported to the GCP center of the clinical research sponsor and the main person in charge of the research project within 24 hours.

8.2 SAEs include：

- Events leading to death
- Events threatening life
- Events leading to urgent need for hospitalization for outpatients or leading to longer hospital stays
- Events that cause persistent dysfunction or disease which cannot be recovered
- Events that cause women in childbearing age to produce congenital malformations or infertility

8.3 ADRs and SAEs Reporting address：

Department of GCP, Chinese Academy of Medical Sciences

Shuting Li

TEL：010-87788495

Email：list@pubem.cicams.ac.cn

10.Management principles

- Lead by the Chinese Academy of Medical Sciences, the main research leader of the center is Director Yexiong Li..
- Researchers at the center are responsible for obtaining approvals from the ethics committee and performing randomization and quality control.
- The researchers must obtain the patient's informed consent and save it before treatment.

11. Lists and contact details for multiple centers

Chinese Academy of Medical Sciences:：

Radiotherapy department：YeXiong Li, Jing Jin, Shulian Wang, Weihu Wang, Yueping Liu; Yongwen Song, Zihao Yu, Hui Fang, Ning Li, Yuan Tang, Xin Wang, Ningning Lu,etc.

Etiology department：Dongxin Lin, Wen Tan, etc

Department of Abdominal Surgery: Zhixiang Zhou, Xinghua Yuan, Haizeng Zhang, Qian Liu, Jianjun Bi, Zhaoxu Zheng, etc.

Internal medicine：Honggang Zhang, Aiping Zhou, Lin Yang, Yihebali Chi, Jinwa Wang, etc.

List of other hospitals: see attached table

12 Research Progress：

Time to start: January 2008

End of patient enrollment: December 2015

Number of participants: 250 cases

13 The principle of randomization

Our center lead the principle of randomization, Patients who meet the enrollment requirements should be filled in by the research unit and then faxed to the Department of Radiation Oncology, Cancer Hospital, Chinese Academy of Medical Sciences, and phone Doctor Jing Jin. Random number table method was used for randomization. After enrolling, Professor Jing Jin will inform the research unit.

Department of Radiation Oncology, Chinese Academy of Medical Sciences: 10-67706153 (fax)

13. Informed consent of patients

- This is a study to compare the efficacy of two treatments for rectal cancer.
- If the rectal tumor invades the rectal wall or has lymph node metastasis, postoperative radiotherapy and chemotherapy is a necessary treatment plan, which can improve the curative effect of operation, reduce the local recurrence rate and improve the survival rate. In recent years, many new and more effective chemotherapeutic drugs, such as oxaliplatin and capecitabine, have emerged on the basis of the original 5-fluorouracil chemotherapy. Previous studies have proved that oxaliplatin combined with capecitabine and 5-fluorouracil is superior to 5-fluorouracil alone in the first-line chemotherapy and adjuvant treatment of colorectal cancer, while capecitabine is easy to take and has the same efficacy as 5-fluorouracil. How to combine radiotherapy with these drugs in order to achieve better curative effect without increasing the adverse reactions is the purpose of our study.
- Your participation in this study is based on the standard treatment plan, which will not affect your normal treatment and curative effect. In addition, it is possible to achieve better curative effect than conventional treatment. This study uses a randomized approach, and you may be treated either with conventional methods or with more aggressive treatments.
- Before treatment, you need to carry out a number of necessary clinical examinations, such as hematology examination, physical examination, imaging examination and tumor biopsy. These examinations are routine examinations and have nothing to do with your participation in clinical research. The purpose of these examinations is to understand the changes of your condition and the adverse reactions of treatment, and give you corresponding treatment when necessary.
- Treatment procedure: the patients were operated first, and then randomly divided into two groups to receive concurrent chemoradiotherapy. Group 1 received oxaliplatin + capecitabine concurrent chemoradiotherapy, capecitabine 1300mg / m2 / D, twice a day, with an interval of 12 hours. Oxaliplatin was infused intravenously once a week. During radiotherapy, it was injected four times in the first, second, fourth and fifth weeks. During injection and 1-2 days before and after injection, attention should be paid to keep warm and avoid cold stimulation. The other group only received capecitabine chemotherapy at the same time. Capecitabine 1600mg / m2 / D was taken orally in two times, with an interval of 12 hours, every day, in the first, second, fourth and fifth weeks of radiotherapy, for a total of 4 weeks. High energy X-ray treatment machine was used to irradiate the pelvic cavity (tumor area and lymph node drainage area). Radiotherapy was performed from week 1 to week 5, with rest on Saturday and Sunday. The treatment time was about 5 weeks. Adjuvant chemotherapy after concurrent chemoradiotherapy takes about half a year. So far, the whole treatment cycle is over.
- During radiotherapy and chemotherapy, there may be nausea, vomiting, diarrhea, fatigue, numbness of fingers and toes, leukopenia and other symptoms. Some reactions are normal treatment reactions, and some may be related to medication. No matter what kind of reaction occurs during the treatment, I hope you can report to your doctor in charge in time. The doctor will make corresponding treatment according to the specific situation to relieve the symptoms.
- After all treatment, you should also conduct regular follow-up to find and solve problems in time. We require you to come to our hospital for follow-up every 3 months within 2 years after the end of all

treatment, which requires you to do some hematological and imaging examinations; those who are more than 2 years and less than 5 years will be followed up every 6 months, the content is the same as above. Regular follow-up is a very important part of tumor treatment. If you can't come to our hospital for reexamination, we may contact you regularly by telephone to understand your current situation.

- If the study is unfavorable to you, or you are unable to comply with the treatment principles, or you are found not to meet the inclusion criteria, the doctor can ask you to withdraw from the study.
- You are willing to participate in this research, and you can withdraw at any time without any explanation. In this case, you must inform your doctor so that your condition can be properly assessed and recorded, and you can continue to receive appropriate treatment.
- If you participate in this research, your records may be provided to our hospital for scientific research and publication, but any personal information about you will be kept confidential. If you have any questions about this study, please contact your doctor.

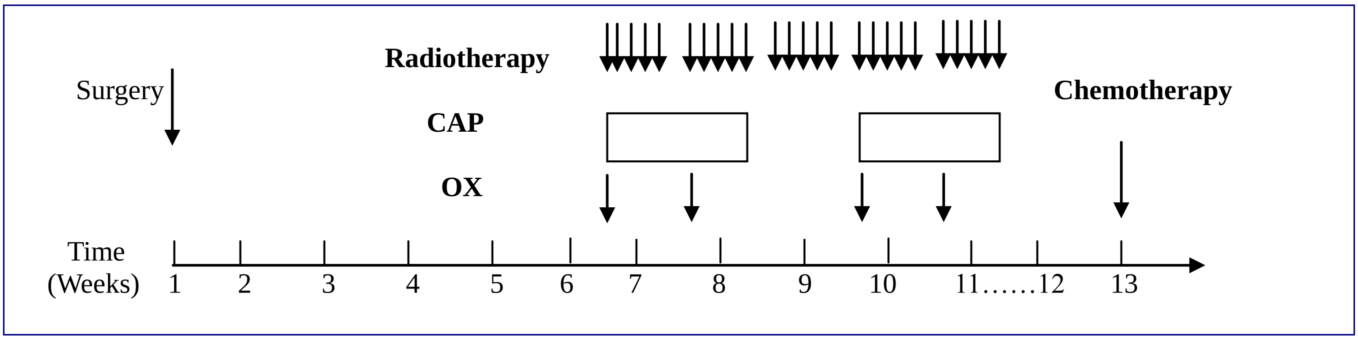

Informed consent of patients

I have been informed of the study of postoperative concurrent chemoradiotherapy + surgery and establishment of molecular marker spectrum. I have made clear the research purpose, procedure, duration, possible efficacy and adverse reactions of postoperative concurrent chemoradiotherapy + basic research. I am willing to participate in this treatment study and understand that I am free to refuse to participate and may withdraw from this treatment study at any time.

If you need more information or help from a doctor, please call 87788122, mobile phone: 13601365130, contact person: Dr. Jin Jing.

Patient signature： ______________ Date： _________________

Signature of researcher：______________ Date： _________________

14. Appendix

14.1 Karnofsky performance score

14.2 Adverse events CTC 3.0

14.3 Clinical stage of rectal cancer (UICC/AJCC，2002）

14.4Quality of life scale（EORTC QOL C30）
