# Supplementary material for: Postoperative Chemoradiotherapy With Capecitabine and Oxaliplatin vs Capecitabine for Stage II to III Rectal Cancer: A Randomized Clinical Trial
Source: JAMA Netw Open. 2021 Nov 30;4(11):e2136116. doi: 10.1001/jamanetworkopen.2021.36116 (PMC8634060; doi:10.1001/jamanetworkopen.2021.36116)
Supplement: Supplement 2. — eTable 1. Completion of Adjuvant Chemoradiotherapy and Chemotherapy eTable 2. Overview of Randomized Clinical Trials of Adding Oxaliplatin to 5-FU– or Capecitabine-Based Chemoradiotherapy for Rectal Cancer in Neoadjuvant or Adjuvant Settings [file jamanetwopen-e2136116-s002.pdf]

Supplemental Online Content

Li N, Zhu Y, Liu LY, et al. Postoperative chemoradiotherapy with capecitabine and oxaliplatin vs capecitabine for stage II to III rectal cancer: a randomized clinical trial. *JAMA Netw Open*. 2021;4(11):e2136116. doi:10.1001/jamanetworkopen.2021.36116

eTable 1. Completion of Adjuvant Chemoradiotherapy and Chemotherapy
eTable 2. Overview of Randomized Clinical Trials of Adding Oxaliplatin to 5-FU– or Capecitabine-Based Chemoradiotherapy for Rectal Cancer in Neoadjuvant or Adjuvant Settings

This supplemental material has been provided by the authors to give readers additional information about their work.

© 2021 Li N et al. *JAMA Network Open.*

eTable 1. Completion of Adjuvant Chemoradiotherapy and Chemotherapy

	CAP-RT n (%)	CAPOX-RT n (%)	*P*
Radiotherapy			*0.02*
Complete as planned	291 (99.0)	284 (96.3)	
Not complete	3 (1.0)	11 (3.7)	
Concurrent chemotherapy			*0.001*
Complete as planned	219 (74.5)	197 (66.8)	
Complete after dose reduction	67 (22.8)	84 (28.5)	
Not complete	8 (2.7)	14 (4.7)	
Chemoradiotherapy			
Complete as planned	218 (74.1)	195 (66.1)	*0.001*
Adjuvant chemotherapy			
Yes	218 (74.1)	221 (74.9)	*0.17*
No	77 (25.9)	73 (25.1)	

CAP, capecitabine; OX, oxaliplatin; RT, radiotherapy.

© 2021 Li N et al. *JAMA Network Open.*

eTable 2. Overview of Randomized Clinical Trials of Adding Oxaliplatin to 5-FU– or Capecitabine-Based Chemoradiotherapy for Rectal Cancer in Neoadjuvant or Adjuvant Settings

Trial (period)	**No.**	**Inclusion criteria**	**Stage**	**R0 (%)**	**Regimen**	**RT**	**Dose (Gy)**	**Adjuvant CT (%)**	**pCR (%)**	**Grade 3/4 toxicity (%)**	**5-year LR (%)**	**5-year DFS (%)**	**5-year OS (%)**
ACCORD12[22] (2005–2008)	598	<12 cm*; T3/4 Nx; distal anterior T2 Nx	T3–4 92.8%; III 72.3%.	94.1	CAP+nRT<br>OX+CAP+nRT	3–4 fields, conventional	45<br>50	5-FU+LV (42.3%)	13.9<br>19.2	10.9<br>25.4	6.1 (3-y)<br>4.4 (3-y)	67.9 (3-y)<br>72.7 (3-y)	87.6 (3-y)<br>88.3 (3-y)
NSABP R-04[24] (2004–2010)	1608	<12 cm*; T3/4 and/or cN+	III 40.7%.	<93	CAP/5FU+nRT<br>OX+CAP/5FU+nRT	Large field	45 Boost 5.4	Not specified	19.1<br>20.9	6.6<br>15.4	12.1<br>11.2	64.2<br>69.2	79.0<br>81.3
STAR-01[25] (2003–2008)	747	<12 cm*; T3/4 and/or cN+	T3–4 96.6%; III 65.3%.	89.7	5FU+nRT<br>OX+5FU+nRT	Conventional or 3D-CRT	50.4	Not specified	16<br>16	8<br>24	NA	62.3<br>67.4	77.6<br>80.4
CAO/ARO-04[23] (2006–2010)	1265	<12 cm*; T3/4 and/or cN+.	T3–4 93.0%; III 71.4%.	90.9	5FU+nRT<br>OX+5FU+nRT	3–4 fields, conventional	50.4	5FU×4 (78%)<br>5FU+OX×8 (78%)	13<br>17	20<br>23	NA	71.2 (3-y)<br>75.9 (3-y)	88.0 (3-y)<br>88.7 (3-y)

© 2021 Li N et al. *JAMA Network Open.*

PETACC-6[26] (2008–2011)	1094	<12 cm*; T3/4 and/or cN+	T3–4 93.2%; III 71.1%.	94.6	CAP+nRT<br>OX+CAP+nRT	3–4 fields, conventional	45–50.4	CAP±OX 4.5 m (74% *vs.* 88%)	11.3<br>13.3	15.1<br>36.7	8.7<br>6.0	71.3<br>70.5	83.1<br>80.1
Present study (2008–2015)	589	pT3/4 and/or pN+	T3–4 90.3%; III 75.4%.	100	CAP+aRT<br>OX+CAP+aRT	IMRT/3D-CRT	50	CAPOX or FOLFOX (75%)	–	28.6<br>38.6	7.1<br>4.7	72.0<br>71.1	82.9<br>82.4

*from the anal verge.

RT, Radiotherapy; CT, chemotherapy; pCR, pathologic complete response; LR, local recurrence; DFS, disease-free survival; OS, overall survival; CAP, capecitabine; OX, oxaliplatin; 5FU, 5-fluorouracil; LV, leucovorin; nRT, neoadjuvant radiotherapy; aRT, adjuvant radiotherapy; NA, not available; CAPOX, capecitabine and oxaliplatin; FOLFOX, fluorouracil, leucovorin, and oxaliplatin.

© 2021 Li N et al. *JAMA Network Open.*
