# Supplementary material for: Postoperative Chemoradiotherapy With Capecitabine and Oxaliplatin vs Capecitabine for Stage II to III Rectal Cancer: A Randomized Clinical Trial
Source: JAMA Netw Open. 2021 Nov 30;4(11):e2136116. doi: 10.1001/jamanetworkopen.2021.36116 (PMC8634060; doi:10.1001/jamanetworkopen.2021.36116)
Supplement: Supplement 3. — Data Sharing Statement [file jamanetwopen-e2136116-s003.pdf]

Data Sharing Statement

Li. Postoperative Chemoradiotherapy With Capecitabine and Oxaliplatin vs Capecitabine for Stage II to III Rectal Cancer. *JAMA Netw Open*. Published November 30, 2021. doi:10.1001/jamanetworkopen.2021.36116

Data
Data available: No
